# The Influence of Organizational and Social Work Environmental Factors on Patient Safety Climate

**DOI:** 10.1155/jonm/3083552

**Published:** 2026-06-30

**Authors:** Alexander Agrell, Susanne Tafvelin, Jens Wahlström, Johan Simonsen Abildgaard, Robert Lundmark

**Affiliations:** ^1^ Department of Psychology, Umeå University, Umeå, Sweden, umu.se; ^2^ Department of Epidemiology and Global Health, Umeå University, Umeå, Sweden, umu.se; ^3^ Copenhagen Business School., Copenhagen, Denmark; ^4^ National Research Center for the Working Environment, Copenhagen, Denmark; ^5^ Department of Health, Education and Technology, Luleå University of Technology, Luleå, Sweden, ltu.se

## Abstract

**Background:**

Patient safety climate (PSC) reflects healthcare professionals’ shared perceptions of how patient safety is prioritized, managed, and enacted within healthcare organizations. While PSC is known to be associated with favorable patient outcomes, empirical evidence on how specific work environment factors shape PSC over time remains limited. Drawing on the job demands–resources theory, this study examines how organizational and social work environment factors relate to PSC in a healthcare setting.

**Methods:**

A two‐wave panel study was conducted within a large healthcare organization in northern Sweden. Survey data were collected from employees across four departments at baseline (T1; *n* = 624) and six months later (T2; *n* = 454). Organizational factors included quantitative demands and role clarity, while social factors included teamwork and quality of leadership. PSC was measured at T2. Structural equation modeling was used to test longitudinal relationships between T1 predictors and PSC at T2.

**Results:**

In the structural model, role clarity (*β* = 0.23, *p* < 0.001) and teamwork (*β* = 0.32, *p* < 0.001) at T1 were positively associated with PSC at T2. Quantitative demands and quality of leadership showed no significant unique associations with PSC when all predictors were included simultaneously. The final model explained 32% of the variance in PSC.

**Conclusions:**

The findings indicate that clearly defined roles and effective teamwork are key antecedents of PSC over time, whereas workload and general leadership quality may be less influential when considered alongside other work environment factors.

**Implications for Nursing Management:**

Efforts to strengthen PSC may benefit from interventions targeting multiple aspects of the work environment, with role clarity and collaborative team processes emerging as particularly promising targets for nursing management.

## 1. Background

Patient safety climate (PSC) reflects healthcare professionals’ attitudes and behaviors toward how patient safety is structured and implemented in healthcare organizations [1–3]. A positive PSC is typically recognized by open communication, mutual trust, engaged leadership, and nonpunitive responses to error. These conditions enable healthcare professionals to report incidents and near misses without risking negative repercussions, thereby enables continuous learning and improvement [4, 5]. At the opposite end, a PSC marked by hierarchical barriers, inadequate management support, or reluctance to discuss mistakes can suppress reporting behaviors and undermine learning and patient safety improvement efforts [6–8].

Recent studies have examined PSC and its association with a number of key constructs such as quality of care [9], demographic and organizational factors [10], disruptive behaviors [11], and leadership [12, 13]. Research has consistently linked a favorable PSC to beneficial clinical outcomes, including reduced rates of medical errors, and over time, a reduced risk of unnecessary deaths of patients due to malpractice [14, 15]. As such, assessing and strengthening the safety climate has become a central strategy in global patient safety efforts [16], a key component of quality improvement frameworks [17], and a vital objective for healthcare institutions striving to provide the highest quality of care [16].

Despite recognition that the work environment strongly influences healthcare quality, the empirical evidence linking work environment factors to PSC remains limited [18]. Thus, there is a lack of understanding of how PSC is influenced by the work environment in healthcare organizations, and consequently how PSC can be managed through work environment improvements.

The job demands–resources (JD‐R) theory is widely used as a framework for examining work environments in healthcare where one of its strengths lies in capturing a wide range of positive and negative work environment factors and their relation to various outcomes, making it well suited to complex healthcare settings [19]. Consequently, the JD‐R theory has been used to effectively explain healthcare staff well‐being, burnout, and work engagement [20]. The JD‐R theory stipulates that work environment factors can be divided into two broad categories: demands (i.e., aspects that require sustained effort and can lead to strain) and resources (i.e., aspects that stimulate growth, help meet demands, and foster engagement) [21]. Furthermore, these work environmental factors can be divided into organizational (e.g., quantitative demands and job task design) and social (e.g., support from leader and social climate) categories [22]. Both the perception of job demands and job resources influences healthcare workers’ work engagement and wellbeing [23, 24]. Hence, given the nature of healthcare work with high and increasing demands and critical but scarce resources [25], JD‐R provides a useful lens for understanding how work environment factors influence outcomes of importance for patients, healthcare professionals, and healthcare organizations [20].

In general, a resourceful work environment in which trust, collaborative efforts, and open communication among staff are promoted can be seen as a vital precondition for identifying risks and preventing adverse events [26, 27]. Empirical evidence indicates that work environments characterized by collegial support, fair management, and participative decision‐making are associated with more positive perceptions of PSC [28–30]. Such work environments encourage staff to voice concerns, share mistakes, and learn collectively without fear of blame, behaviors fundamental to sustaining a strong safety climate [31]. In contrast, a poor work environment, characterized by role ambiguity, limited autonomy, high job demands, and interpersonal conflict, is associated with a poorer PSC, lower engagement in safety behaviors, and an increased risk of human error and underreporting of safety incidents [32–34]. Thus, both organizational and social aspects of a work environment that includes high demands and a lack of resources can be expected to negatively affect PSC, and ultimately patient safety.

Using a two‐wave panel sample, the aim of our study is to examine the relation between both organizational (quantitative demands and role clarity) and social (teamwork and leadership) work environmental factors [21] and PSC. From a JD‐R perspective, these can also be considered as demands (quantitative demands) and resources (role clarity, teamwork, and leadership) [35, 36] in healthcare settings that can potentially support or undermine PSC, and thus ultimately patient safety [32, 37].

Among healthcare professionals, high quantitative demands have been associated with increased strain and reduced safety performance [38, 39], which may negatively influence perceptions of patient safety culture. We, therefore, hypothesize the following:

 Hypothesis 1: Quantitative demands will be negatively related to PSC.
 Role clarity among healthcare professionals has been associated with improved team functioning, coordination, and safer care practices, whereas a lack of role clarity may increase the risk of errors and reduce effectiveness in healthcare teams [40–42].
 Hypothesis 2: Role clarity will be positively related to PSC.
 Effective teamwork in healthcare settings has been found to improve communication, coordination, and safer care practices, and has thereby been identified as a core component of patient safety culture [31, 43].
 Hypothesis 3: Teamwork will be positively related to PSC.
 Finally, the quality of leadership has been associated with shaping safety climate, influencing staff attitudes, and promoting safer care practices, and has been shown to significantly predict safety climate in healthcare settings [12, 29].
 Hypothesis 4: Quality of leadership will be positively related to PSC.

Our study contributes by examining work environment antecedents to PSC, thereby widening the scope of what matters to make patient care safe. It also moves beyond the cross‐sectional design of most previous studies, reducing the risk for common‐method bias [34]. By simultaneously examining several factors in the same model, our study also provides insights into the relative importance of these factors for PSC. From that, the findings from our study can help focus the direction of future efforts to improve patient safety, especially in organizations with sparse resources for implementing improvement efforts [33].

## 2. Materials and Methods

### 2.1. Study Design

The study was carried out within a healthcare organization in northern Sweden. The participating organization has a workforce of more than 10,000 employees, the vast majority working in healthcare professions. In November 2021 (T1, baseline), all 1,299 employees across four departments of the organization—a surgical center, a laboratory unit, a habilitation service, and a primary care center—were invited to participate in a survey. These departments were chosen in collaboration with the HR department to represent diverse professional roles, geographical areas, and both specialized and general healthcare services within the organization. Data collection for T1 was open for four weeks, during which five reminders were distributed to employees who had not yet responded. In total, 624 employees completed the questionnaire at T1, corresponding to a response rate of 48%. Six months later, a follow‐up survey (T2) was distributed to those who had participated in T1. A total of 454 employees also responded at T2, resulting in a follow‐up response rate of 73%. Table 1 presents demographic and professional characteristics of the respondents, including gender, age, educational level, profession, and tenure within the organization at T1. The same characteristics of the sample at T2 are provided in as an appendix (see Table A1).

**TABLE 1:** Characteristics of respondents at T1.

Characteristics *N* (%)	Surgery center	Laboratory	Habilitation center	Primary care	Total
Sex					
Female	165 (80.5%)	157 (78.1%)	93 (90.3%)	98 (85.2%)	513 (82.2%)
Male	38 (18.5%)	42 (20.9%)	9 (8.7%)	17 (14.8%)	106 (17.0%)
Other/missing	2 (1.0%)	2 (1.0%)	1 (1.0%)	—	5 (0.8%)
Age mean (sd)	41.5 (12.5)	46.3 (12.0)	49.1 (9.5)	47.4 (11.2)	45.4 (11.9)
Degree					
Primary school	1 (0.5%)	1 (0.5%)	1 (1.0%)	1 (0.9%)	4 (0.6%)
High school	49 (23.9%)	20 (10.0%)	4 (3.9%)	27 (23.5%)	100 (16.0%)
University	144 (70.2%)	147 (73.1%)	96 (93.2%)	86 (74.8%)	473 (75.8%)
PhD	9 (4.4%)	33 (16.4%)	1 (1.0%)	1 (0.9%)	44 (7.0%)
Missing	2 (1.0%)	—	1 (1.0%)	—	3 (0.5%)
Occupation					
Nurse	81 (39.5%)	12 (6.0%)	1 (1.0%)	34 (29.6%)	128 (20.5%)
Assistant nurse	44 (21.5%)	4 (2.0%)	—	22 (19.1%)	70 (11.2%)
Physician	34 (16.6%)	26 (12.9%)	—	10 (8.7%)	70 (11.2%)
Psychologist/counselor	1 (0.5%)	—	38 (36.9%)	7 (6.1%)	46 (7.4%)
Manager	11 (5.4%)	18 (9.0%)	6 (5.8%)	11 (9.6%)	46 (7.4%)
Medical secretary	17 (8.3%)	7 (3.5%)	—	7 (6.1%)	31 (5.0%)
Care administrator	7 (3.4%)	7 (3.5%)	8 (7.8%)	11 (9.6%)	33 (5.3%)
Physiotherapist	2 (1.0%)	—	9 (8.7%)	9 (7.8%)	20 (3.2%)
Biomedical analyst/lab technician	—	127 (63.2%)	—	—	127 (20.4%)
Occupational therapist	1 (0.5%)	—	18 (17.5%)	3 (2.6%)	22 (3.5%)
Other	7 (3.4%)	—	23 (22.3%)	1 (0.9%)	31 (5.0%)
Years in organization					
< 3 years	43 (21.0%)	29 (14.4%)	18 (17.5%)	20 (17.4%)	110 (17.6%)
3–13 years	92 (44.9%)	85 (42.3%)	40 (38.8%)	46 (40.0%)	263 (42.1%)
> 13 years	70 (34.1%)	86 (42.8%)	45 (43.7%)	48 (41.7%)	249 (39.9%)
Missing	—	1 (0.5%)	—	1 (0.9%)	2 (0.3%)

### 2.2. Organizational Factors

#### 2.2.1. Quantitative Demands

Participants’ subjective assessment of the degree of quantitative demands at their workplace was measured using the four‐item subscale *Quantitative Demands* within the Danish Psychosocial and Work Environment Questionnaire (DPQ) [44]. Responses were given on a five‐point Likert scale from 1 (“*never/almost never*”) to 5 (“*always*”). An example item is “Do you get behind with your work?” Scale reliability in the present sample at T1 was *ω* = 0.83.

#### 2.2.2. Role Clarity

Participants’ subjective assessment of their role clarity at their workplace was measured using the four‐item subscale *Role Clarity* within the DPQ [44]. Responses were given on a five‐point Likert scale from 1 (*“strongly disagree”*) to 5 (*“strongly agree”*). An example item is “Do you know exactly what is expected of you at work?” Scale reliability in the present sample at T1 was *ω* = 0.81.

### 2.3. Social Factors

#### 2.3.1. Teamwork

Participants’ subjective assessment of the degree to which employees perceive collaboration and mutual support among coworkers was measured using the four‐item subscale *Cooperation between colleagues within teams, departments, or groups* within the DPQ [44]. Responses were given on a five‐point Likert scale from 1 (*“strongly disagree”*) to 5 (*“strongly agree”*) with the additional option “I don’t have any colleagues.” An example item is “Do you and your colleagues help each other if someone has too much to do?”. Scale reliability in the present sample at T1 was *ω* = 0.84.

#### 2.3.2. Quality of Leadership

Participants’ subjective assessment of the quality of leadership by their immediate supervisor was measured using the four‐item subscale *Quality of Leadership* within the DPQ [44]. Responses were given on a five‐point Likert scale from 1 (*“strongly disagree”*) to 5 (*“strongly agree”*) with the additional option “I don’t have a manager.” An example item is “Does your immediate supervisor give high priority to the wellbeing of employees in the workplace?”. Scale reliability in the present sample at T1 was *ω* = 0.89.

### 2.4. Outcome

#### 2.4.1. PSC

Participants’ subjective assessment of the PSC of their workplace was measured using the seven‐item subscale *Safety Climate* within the Safety Attitudes Questionnaire (SAQ) [45]. Responses were given on a five‐point Likert scale from 1 (*“strongly disagree”*) to 5 (*“strongly agree”*). An example item is “I would feel perfectly safe being treated here as a patient.” Scale reliability in the present sample at T2 was *ω* = 0.81.

#### 2.4.2. Control Variables

Consistent with prior research, we controlled for *age, gender*, and *tenure*, as these demographic variables are widely used in organizational research and have been shown to relate systematically to individual attitudes and behaviors and may confound relationships among study variables of interest [46–48].

#### 2.4.3. Data Analysis

All calculations for this study were made using Mplus Version 8.10 [49]. For the structural equation modeling (SEM), we applied the robust full information maximum likelihood estimator (MLR) to account for missing data and potential non‐normality of the data [50]. For all analyses, we used a significance level of *p* < 0.05.

We used a three‐step approach:

 Step 1: Latent means and standard deviations were estimated for all variables to describe their central tendency and variability at the latent level. McDonald’s omega (*ω*) coefficients were estimated to assess the internal consistency reliability of the latent variables [51].
 Step 2: We estimated the measurement model using confirmatory factor analysis (CFA) to evaluate the factorial validity of the latent constructs [52]. Model fit was assessed using the root mean square error of approximation (RMSEA), the comparative fit index (CFI), the Tucker–Lewis index (TLI), and the standardized root mean residual (SRMR). Following conventional guidelines, RMSEA and SRMR values < 0.08 and CFI and TLI values > 0.90 were considered indicative of acceptable model fit [52].
 Step 3: Based on the established measurement model, we estimated a structural model in which PSC at T2 was regressed on quantitative demands, role clarity, teamwork, and quality of leadership at T1. In a subsequent step, the control variables age, gender, and tenure were added to the model to examine whether their inclusion improved model fit and whether any of these covariates had statistically significant associations with the variables in the model.

## 3. Results

**FIGURE 1:**
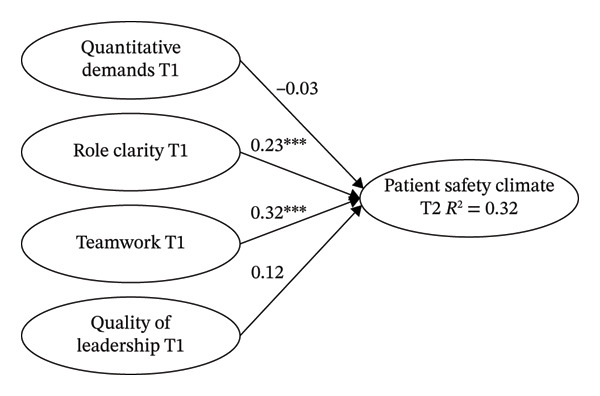
Structural equational model. ^∗∗∗^ *p* < 0.001.

 Step 1: Descriptive statistics, correlations, and reliability
 Descriptive statistics, bivariate correlations, and McDonald’s omega (*ω*) reliability estimates can be found in Table 2. Most correlations were statistically significant and in the expected directions. Notably, none of the control variables showed significant correlations with any of the other variables. The omega reliability coefficients ranged from 0.81 to 0.89, thus indicating good internal consistency across the measures as long as the measurement model (see Step 2) is acceptable [51].
 Step 2: Measurement model
 The CFA demonstrated an acceptable fit to the data (*χ* ^2^(220) = 522.829, *p* < 0.001, RMSEA = 0.047, 90% CI [0.042, 0.052], CFI = 0.937, TLI = 0.928, SRMR = 0.054). Therefore, we proceeded to examine the structural model.
 Step 3: Structural model
 In the SEM‐model, see Figure 1, employees’ perceptions of quantitative demands did not show any significant relationship with PSC at T2, therefore rejecting Hypothesis 1.

Furthermore, role clarity at T1 (*β* = 0.23, *p* < 0.001) and teamwork at T1 (*β* = 0.32, *p* < 0.001) were positively related to PSC at T2 supporting our Hypotheses 2 and 3. These findings indicate that clearly defined roles and effective collaboration among employees are important antecedents of PSC.

**TABLE 2:** Latent means, standard deviations, latent variable correlations, and scale reliabilities (omega).

	M	SD	QD T1	RC T1	TW T1	QoL T1	PSC T2	Age	Tenure	Gender
Quantitative demands T1	2.67	0.68	*ω* = 0.83	—	—	—	—	—	—	—
Role clarity T1	3.61	0.68	−0.27^∗∗∗^	*ω* = 0.81	—	—	—	—	—	—
Teamwork T1	3.96	0.63	−0.19^∗∗∗^	0.49^∗∗∗^	*ω* = 0.84	—	—	—	—	—
Leadership T1	3.87	0.79	−0.24^∗∗∗^	0.50^∗∗∗^	0.53^∗∗∗^	*ω* = 0.89	—	—	—	—
Patient safety climate T2	4.10	0.45	−0.18^∗∗^	0.44^∗∗∗^	0.51^∗∗∗^	0.42^∗∗∗^	*ω* = 0.81	—	—	—
Age	45.38	11.93	−0.09	0.09	−0.11	0.06	0.08	—	—	—
Tenure	13.35	12.29	0.08	−0.08	0.03	−0.08	0.04	0.66^∗∗∗^	—	—
Gender	1.17	0.38	0.04	−0.03	0.03	0.06	−0.01	−0.04	−0.11^∗∗^	—

*Note: N* = 624. Reliabilities (omega) are presented on the diagonal. Gender: 1 = female, 2 = male. T1 = Time 1 (baseline), T2 = Time 2 (follow‐up).

Abbreviations: M, mean; PSC, patient safety climate; QD, quantitative demands; QoL, leadership/quality of leadership; RC, role clarity; SD, standard deviation.

^∗∗^ *p* < 0.01.

^∗∗∗^ *p* < 0.001.

Lastly, quality of leadership at T1 was not significantly associated with PSC at T2. Thus, Hypothesis 4 was rejected.

Although quantitative demands and quality of leadership at T1 were significantly correlated with PSC at T2 at the bivariate level (Table 2), neither variable showed a statistically significant unique association with PSC when included simultaneously with the other predictors in the structural model.

Based on recommendations of Becker et al. [53], we compared a model including control variables, which in this study were age, gender, and tenure, with a model in which these controls were removed. None of the control variables showed any significant association with PSC and did not alter the association of the independent variables and PSC in any conspicuous way. Additionally, when adding control variables, the model showed a deterioration of model fit. Therefore, in the final model, the control variables were excluded.

Altogether, the structural model explained 32% of the variance in PSC, suggesting an important role of task‐related clarity and collaborative processes in shaping employees’ perceptions of PSC over time.

## 4. Discussion

The aim of the current study was to examine how organizational (quantitative demands and role clarity) and social (teamwork, quality of leadership) factors in the work environment are related to PSC over time in a healthcare setting. In our two‐wave panel design, role clarity and teamwork were significant predictors of PSC six months later, whereas quantitative demands and quality of leadership were not significant associated with PSC in the final model.

Among the organizational factors, only role clarity predicted PSC, which supports the notion that clear expectations and well‐defined responsibilities are central to delivering safe, high‐quality healthcare [54]. Ambiguity and uncertainty about one’s role may hinder coordination, complicate decision‐making, and increase uncertainty during routine and critical tasks which may lead to mistakes and inefficient use of time and resources [55]. From a managerial perspective, this suggests that ensuring clarity around duties, reporting structures, and task boundaries can strengthen foundational conditions for a positive PSC. In contrast, quantitative demands did not show a significant relationship with PSC. Although heavy workload is often highlighted as something that can jeopardize patient safety [56], it is possible that a high workload has become a normalized condition and healthcare professionals do not compromise when it comes to views on patient safety because of a high workload.

Among the social factors, teamwork was a strong predictor of PSC, emphasizing the importance of collaboration, mutual support, and shared situational awareness in nursing and interprofessional teams. This aligns with research demonstrating that cohesive workgroups are better equipped to detect risks, communicate concerns, and adapt to unexpected situations, all essential components of a strong safety climate [57]. Unexpectedly, leadership, which at a bivariate level correlated (*r* = 0.42) with PSC, did not show a significant association with PSC when analyzed in the final model together with the other antecedents. Given the extensive literature linking leadership to safety culture and safety outcomes, this finding warrants further attention. Several explanations are possible. The leadership measure assessed general supervisory behaviors rather than leadership behaviors specifically focused on safety practices, which may be more influential for PSC. Leadership effects may also operate indirectly through relational mechanisms such as teamwork or communication quality in the healthcare units. Or it can simply be that teamwork is more influential than leadership when it comes to handling day‐to‐day situations where effective team coordination minimizes the risk for adverse events and strengthens shared perception of safety in the team. Lastly, much of the empirical evidence is based on cross‐sectional study designs, and our study has a more robust study design which may contribute to a more trustworthy estimate of the relationship. However the case may be, this highlights the need for future studies to investigate whether more nuanced or safety‐oriented leadership constructs to better capture the leadership and PSC relationship or if other aspects, such as teamwork, are more important.

## 5. Practical Implications

The findings highlight two actionable implications for nursing management. First, healthcare organizations should promote role clarity by ensuring that responsibilities, expectations, and task boundaries are clearly defined. This can be done through clear job descriptions, structured task delegation, consistent communication, and consistently putting an effort into removing hindrances that are making the tasks and/or decision‐making processes unclear for employees [40].

Second, strengthening the collaborative climate within nursing teams is likely to lead to benefits for patient safety. Interventions targeting teamwork, such as structured handovers, multidisciplinary huddles, peer‐support practices, and after‐action reviews, may amplify the psychosocial resources in the nursing teams and thereby support patient safety and high‐quality care. Such interventions may, for example, involve using a systematic and participatory way of identifying, prioritizing, action planning, and evaluating such areas of improvement [58].

## 6. Limitations and Future Research

Even though our study has robust elements such as a two‐wave data collection, simultaneous examination of multiple antecedents, and practical relevance for healthcare organizations in obtaining and/or retaining a good PSC, the results should be viewed and interpreted in light of its limitations. The study was conducted within four departments in a single healthcare organization, which may limit the generalizability of our findings to the wider healthcare sector. Additional studies should be conducted to determine if these findings are consistent across other healthcare specialties as well as in other countries’ healthcare systems. Future research is needed to replicate and extend these findings in larger and more heterogeneous samples to enhance generalizability across healthcare settings.

In addition, data collection occurred at the end of 2021 until mid‐2022, during a period when healthcare services were still experiencing prolonged strain related to the COVID‐19 pandemic. This context may have influenced both the perceived work environment and PSC. For example, US healthcare workers reported a decline in teamwork and patient safety culture metrics during the pandemic [59], and since the teamwork aspect is central to the results in this study, the potential effect of the pandemic or its aftermath on this construct should be considered. Something that speaks against this though is the relatively high reported levels of both teamwork and PSC.

Furthermore, our model included only four predictors and, even though they are theoretically meaningful, PSC is likely shaped by a broader range of organizational and psychosocial factors that were not captured in the present study. Future research may also further explore the role of leadership. Given the absence of a leadership effect in the current study, future studies should examine safety‐specific leadership behaviors and explore whether leadership influences PSC indirectly through teamwork, psychological safety, or communication pathways.

Finally, to strengthen the robustness of conclusions, future research should investigate the relationship between PSC and objective measures of patient safety outcomes to clarify whether PSC acts as a mediator.

## 7. Conclusions

The current study contributes to the understanding of how organizational and social work environment factors relate to PSC by demonstrating that some, but not all, predictors exert a unique influence on this outcome. Specifically, when included in the same model, only two of the factors, role clarity and teamwork, were statistically significantly associated with PSC. Quantitative demands and leadership, although correlated with PSC at the bivariate level, did not retain significance in the full structural model. Overall, the findings emphasize that both organizational and social conditions matter for patient safety but that clarity in roles and strong team collaboration may be particularly critical when multiple work environment factors are considered simultaneously.

## Funding

This work was supported by Företagsforskarskolan för Samverkan och Innovation, Umeå University (50%) and by Region Västerbotten (50%).

## Disclosure

All materials in the paper are original and have not been presented previously. The final manuscript was reviewed and approved by the authors, who take full responsibility for its content.

## Ethics Statement

Ethical approval for this study was granted by the Swedish Ethical Review Authority under Grant 2021‐03877.

## Consent

Informed consent was obtained from all individual participants included in the study.

## Conflicts of Interest

The authors declare no conflicts of interest.

## Data Availability

The data that support the findings of this study are available on request from the corresponding author. The data are not publicly available due to privacy or ethical restrictions.

## Appendix A

Characteristics of respondents at T2.

**Table A1:** Characteristics of respondents at T2.

Characteristics *N* (%)	Surgery center	Laboratory	Habilitation center	Primary care	Total
Sex					
Female	105 (79.5%)	122 (76.7%)	74 (90.2%)	71 (87.7%)	372 (81.9%)
Male	26 (19.7%)	36 (22.6%)	8 (9.8%)	10 (12.3%)	80 (17.6%)
Other/missing	1 (0.8%)	1 (0.6%)	—	—	2 (0.4%)
Age mean (sd)	44.3 (12.5)	45.7 (11.8)	49.3 (9.43)	47.5 (11.2)	46.3 (11.6)
Degree					
Primary School	1 (0.8%)	—	1 (1.2%)	1 (1.2%)	3 (0.7%)
High school	29 (22.0%)	16 (10.1%)	4 (4.9%)	21 (25.9%)	70 (15.4%)
University	95 (72.0%)	114 (71.7%)	75 (91.5%)	59 (72.8%)	343 (75.6%)
PhD	6 (4.5%)	29 (18.2%)	1 (1.2%)	—	36 (7.9%)
Missing	1 (0.8%)	—	1 (1.2%)	—	2 (0.4%)
Occupation					
Nurse	53 (40.2%)	10 (6.3%)	1 (1.2%)	20 (24.7%)	84 (18.5%)
Assistant nurse	23 (17.4%)	2 (1.3%)	—	15 (18.5%)	40 (8.8%)
Physician	20 (15.2%)	20 (12.6%)	—	6 (7.4%)	46 (10.1%)
Psychologist/counselor	1 (0.5%)	—	30 (36.6%)	7 (8.6%)	38 (8.4%)
Manager	8 (6.1%)	12 (7.5%)	4 (4.9%)	10 (12.3%)	34 (7.5%)
Medical secretary	13 (9.8%)	4 (2.5%)	—	6 (7.4%)	23 (5.1%)
Care administrator	6 (4.5%)	5 (3.1%)	8 (9.8%)	10 (12.3%)	29 (6.4%)
Physiotherapist	2 (1.5%)	—	7 (8.5%)	4 (4.9%)	13 (2.9%)
Biomedical analyst/lab technician	—	106 (66.7%)	—	—	106 (23.3%)
Occupational therapist	1 (0.5%)	—	14 (17.1%)	2 (2.5%)	17 (3.7%)
Other	5 (3.8%)	—	18 (22.0%)	1 (1.2%)	24 (5.3%)
Years in organization					
< 3 years	19 (14.4%)	22 (13.8%)	14 (17.1%)	16 (19.8%)	71 (15.6%)
3–13 years	60 (45.5%)	72 (45.3%)	33 (40.2%)	31 (38.3%)	196 (43.2%)
> 13 years	53 (40.2%)	64 (40.3%)	35 (42.7%)	33 (40.7%)	185 (40.7%)
Missing	—	1 (0.6%)	—	1 (1.2%)	2 (0.4%)
